# LncRNA SUMO1P3 acts as a prognostic biomarker and promotes hepatocellular carcinoma growth and metastasis

**DOI:** 10.18632/aging.202921

**Published:** 2021-04-26

**Authors:** Shu Hu, Jiancheng Liu, Shuying Feng, Yue Wang, Hongchao Liu

**Affiliations:** 1 Medical College, Henan University of Science and Technology, Luoyang 471003, Henan, China

**Keywords:** lncRNA SUMO1P3, hepatocellular carcinoma, proliferation, invasion

## Abstract

Long noncoding RNAs (lncRNAs) are involved in the progression of various cancers, including hepatocellular carcinoma (HCC). However, the biological functions of lncRNA small ubiquitin-like modifier 1 pseudogene 3 (SUMO1P3) and the underlying mechanisms remain unclear. In this study, we revealed that SUMO1P3 expression was enhanced in HCC tissues and cell lines, positively associating with tumor size and number, poor differentiation, lymphatic and distant metastasis, TNM stage, and poor prognosis in HCC patients. *In vitro* assays showed that SUMO1P3 depletion reduced HCC cell viability and proliferation by hindering cyclin D1 expression and Akt phosphorylation. SUMO1P3 knockdown induced HCC cell apoptosis, as indicated by increased Bax and cleaved caspase-3 expression and the decreased Bcl-2 level. SUMO1P3 silencing suppressed HCC cell migration and invasion by increasing epithelial marker E-cadherin expression and decreasing mesenchymal marker vimentin expression, as well as reducing matrix metalloproteinase (MMP)-2 and MMP-9 levels. Consistently, SUMO1P3 depletion in HCC cells retarded tumor growth and lung metastasis *in vivo*. Overall, these results supported the applicability of SUMO1P3 as a useful predictor of HCC prognosis and a potential therapeutic target for HCC patients.

## INTRODUCTION

Hepatocellular carcinoma (HCC) is a highly predominant malignancy with high mortality, and its incidence has continued to increase worldwide [1]. HCC is the fourth most frequent cancer and the third cancer-related death in China, with 422,100 deaths in 2015 [2]. HCC is a complex process involving a variety of environmental and genetic alterations. Despite recent improvements in HCC therapy, such as chemotherapy, radiotherapy, and surgical resection, HCC prognosis remains unsatisfactory owing to the recurrence and/or metastasis [3, 4]. According to previous reports, the 5-year survival rate remains low and unsatisfied for HCC patients [4, 5]. Thus, identifying the underlying mechanisms of HCC tumorigenesis and exploring therapeutic targets for HCC are in urgent need.

Long noncoding RNAs (lncRNAs) belong to a subgroup of noncoding RNA transcripts (>200 nucleotides) known as key regulators for cellular processes at the transcriptional or post-transcriptional level [6, 7]. Growing evidence indicates that lncRNAs act as tumor supporters or tumor suppressors by controlling cellular processes, such as differentiation, proliferation, invasion, and metastasis [6–9]. Dysregulation of some lncRNAs has been verified in various cancers, such as lung cancer, breast cancer, gastric cancer, bladder cancer, and HCC [10–15]. LncRNA small ubiquitin-like modifier 1 pseudogene 3 (SUMO1P3) is originally recognized as a potential biomarker in the diagnosis of gastric cancer [12]. Zhan et al. [13] reported that SUMO1P3 upregulation predicts the poor prognosis of bladder cancer patients and promotes the cell proliferation, migration, invasion, and apoptosis resistance of bladder cancer cells. However, the biological roles and underlying mechanisms of SUMO1P3 in HCC are largely unknown.

Here, we found that the expression of SUMO1P3 was remarkably increased in HCC tissues and cell lines compared to adjacent noncancerous tissues and the normal hepatocyte cell line, respectively. SUMO1P3 enhancement was positively related to tumor size and number, poor differentiation, lymphatic and distant metastasis, TNM stage, and poor outcome of HCC patients. The results of *in vitro* experiments indicated that SUMO1P3 silencing in HCC cell inhibited proliferation, migration, and invasion but promoted apoptosis; however, SUMO1P3 overexpression in normal liver cells showed the opposite effects. Consistently, the *in vivo* findings revealed that SUMO1P3 knockdown hindered HCC growth and lung metastases. Overall, these data provided convincing evidences for the utilization of SUMO1P3 as a promising biomarker for HCC prognosis and as a potential target for HCC therapy.

## RESULTS

### Increased SUMO1P3 expression in human HCC tissues and cell lines is associated with poor prognosis of HCC patients

**Figure 1:**
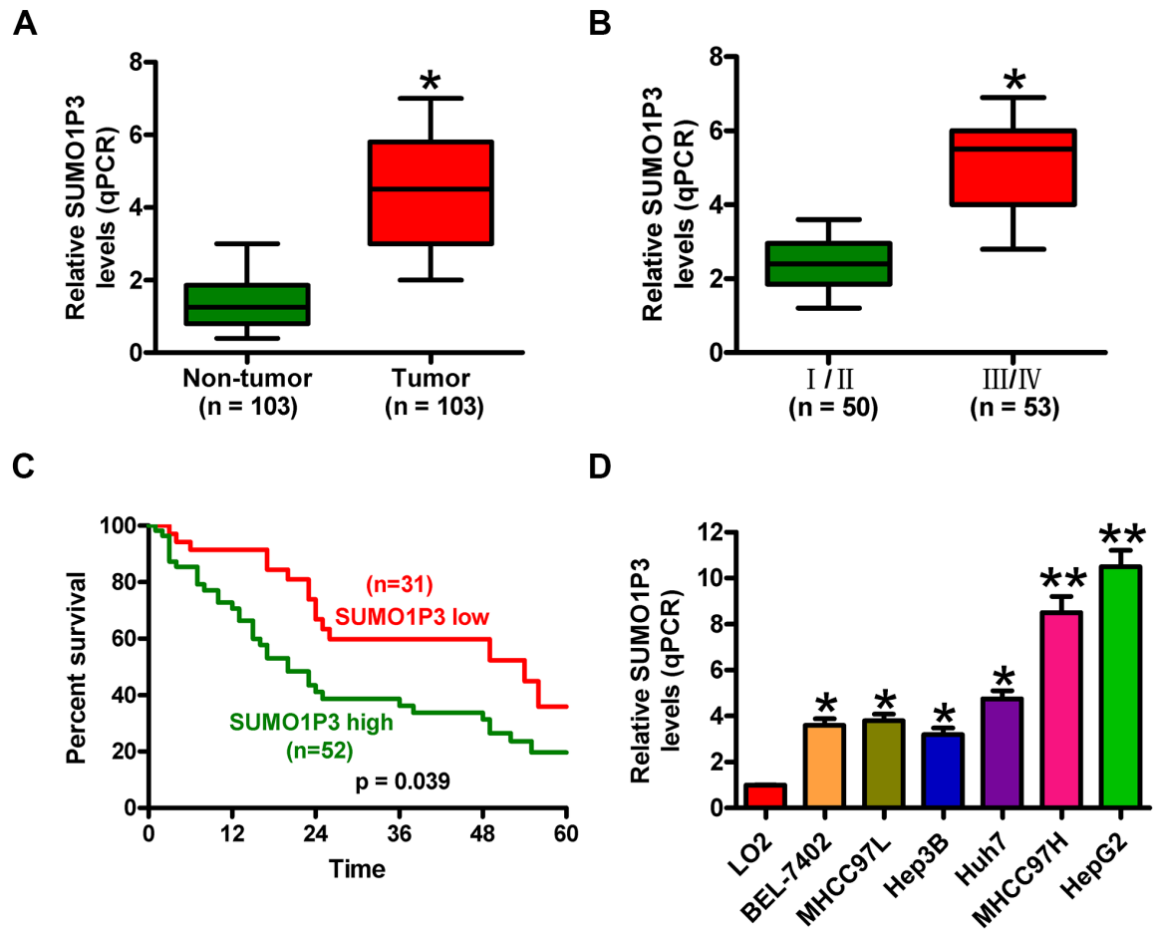
**SUMO1P3 was upregulated in HCC tissues and cell lines and associated with poor survival in HCC patients.** (**A**) qPCR assay was performed to detect SUMO1P3 expression in 103 pairs of HCC tissues and adjacent noncancerous tissues. GAPDH was used as the endogenous control. (**B**) Association between SUMO1P3 differential expression and TNM stage. The SUMO1P3 expression was normalized to GAPDH. (**C**) Kaplan–Meier curves and log-rank test for the 5-year survival rate of HCC patients with high (n=51) or low (n=32) expression of SUMO1P3. (**D**) qPCR analyses of SUMO1P3 expression in HCC cell lines (BEL-7402, MHCC97L, Hep3B, Huh7, MHCC97H, and HepG2) and the normal hepatocyte cell line LO2. GAPDH was used as the endogenous control. All data are represented as the mean ± SD of three replicates. *P < 0.05 vs. Non-tumor or I/II or LO2 group; **P < 0.01 vs. LO2 group.

SUMO1P3 expression was measured in 103 pairs of HCC tissues and adjacent noncancerous samples via qPCR assays. Figure 1A shows that SUMO1P3 was increased in HCC tissues compared to matched adjacent noncancerous tissues. Clinically, SUMO1P3 upregulation was closely associated with tumor size and number, differentiation, lymphatic and distant metastases, and TNM stage (Table 1 and Figure 1B) but not with other characteristics, such as age and gender of HCC patients (Table 1). Furthermore, the patients with SUMO1P3 high-expression had higher 5-year survival rate than that in SUMO1P3 low-expression patients (Figure 1C). In addition, SUMO1P3 was highly expressed in six HCC cell lines, namely, BEL-7402, MHCC97L, Hep3B, Huh7, MHCC97H, and HepG2, compared with the normal hepatocyte cell line LO2 (Figure 1D). MHCC97H and HepG2 cell lines, which exerted the highest expression of SUMO1P3, were selected for subsequent studies. These results indicated that upregulated SUMO1P3 may be used as prognostic markers of HCC.

**Table 1:** The correlation of SUMO1P3 expression with clinicopathological parameters of HCC patients.

**Variables**	**Clinicopathological parameters**	**Case No.** **(n = 103)**	**SUMO1P3 expression**		**P value**
			**High (n = 54)**	**Low (n = 49)**	
Gender	Male	64	28	26	0.421
	Female	39	26	23	
Age (years)	< 65	76	40	36	0.729
	≥ 65	27	14	13	
Size (cm)	< 5	50	19	31	0.002*
	≥ 5	53	35	18	
Differentiation	Well/ Moderate	67	30	37	0.041*
	Poor	36	24	12	
Tumor number	Multiple	76	44	32	0.038*
	Single	27	10	17	
Lymphatic metastasis	N0	42	25	36	0.022*
	N1/ N2	61	28	14	
Distant metastasis	M0	45	18	27	0.016*
	M1	58	36	22	
TNM stage	I/ II	50	19	31	0.002*
	III/ IV	53	35	18	

*P < 0.05.

### SUMO1P3 overexpression empowers the malignant characteristics of normal liver cells

**Figure 2:**
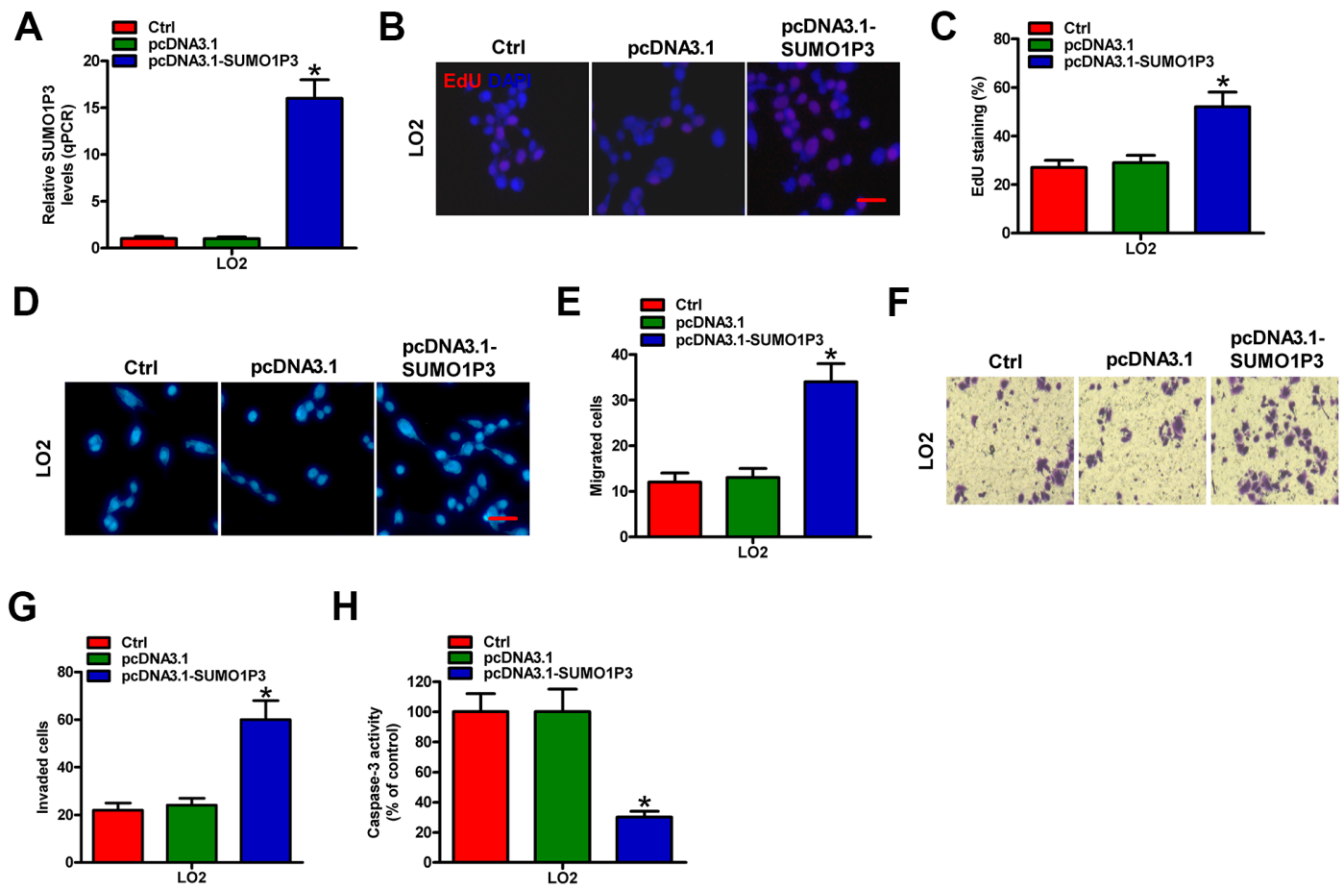
**SUMO1P3 upregulation endowed the malignancies of normal liver cells.** LO2 cells were not transfected (control) or transfected with pcDNA3.1-SUMO1P3 or pcDNA3.1 plasmids. (**A**) The expression of SUMO1P3 was detected by qPCR assays. GAPDH was used as the endogenous control. (**B**) EdU staining was used to detect cell proliferation. Scale bar: 5 μm. (**C**) Percentage of EdU-positive staining in (**B**). Transwell assays were performed to evaluate the migration (**D**) and invasion (**F**) of MHCC97H and HepG2 cells. 100 × magnification in (**F**). The numbers of migrated (**E**) and invaded (**G**) cells were calculated. (**H**) LO2 cells subjected to 24 h of serum starvation were untransfected (control) or transfected with pcDNA3.1-SUMO1P3 or pcDNA3.1 plasmids. Caspase-3 activity was detected by using a commercial kit. All data are represented as the mean ± SD of three replicates. *P < 0.05, **P < 0.01 vs. Ctrl or pcDNA3.1 group.

To address the effects of SUMO1P3 upregulation on normal liver cells, LO2 cells were untransfected (control) or transfected with pcDNA3.1-SUMO1P3 or pcDNA3.1 plasmids. As depicted in Figure 2A, pcDNA3.1-SUMO1P3 plasmid transfection markedly enhanced SUMO1P3 expression in LO2 cells compared with pcDNA3.1-transfected and control cells. SUMO1P3 overexpression promoted LO2 cell proliferation as shown by the elevated EdU incorporation (Figure 2B, 2C). Ectopic expression of SUMO1P3 dramatically increased the migration of LO2 cells compared with pcDNA3.1-transfected or control cells (Figure 2D, 2E). Similarly, cell invasion was markedly suppressed by SUMO1P3 knockdown (Figure 2F, 2G). Moreover, caspase-3 activity was reduced in serum-starved LO2 cells with SUMO1P3-overexpression (Figure 2H). These results illuminated that SUMO1P3 overexpression resulted in the malignant transition of normal liver cells.

### SUMO1P3 depletion inhibits the proliferation of HCC cells *in vitro*

**Figure 3:**
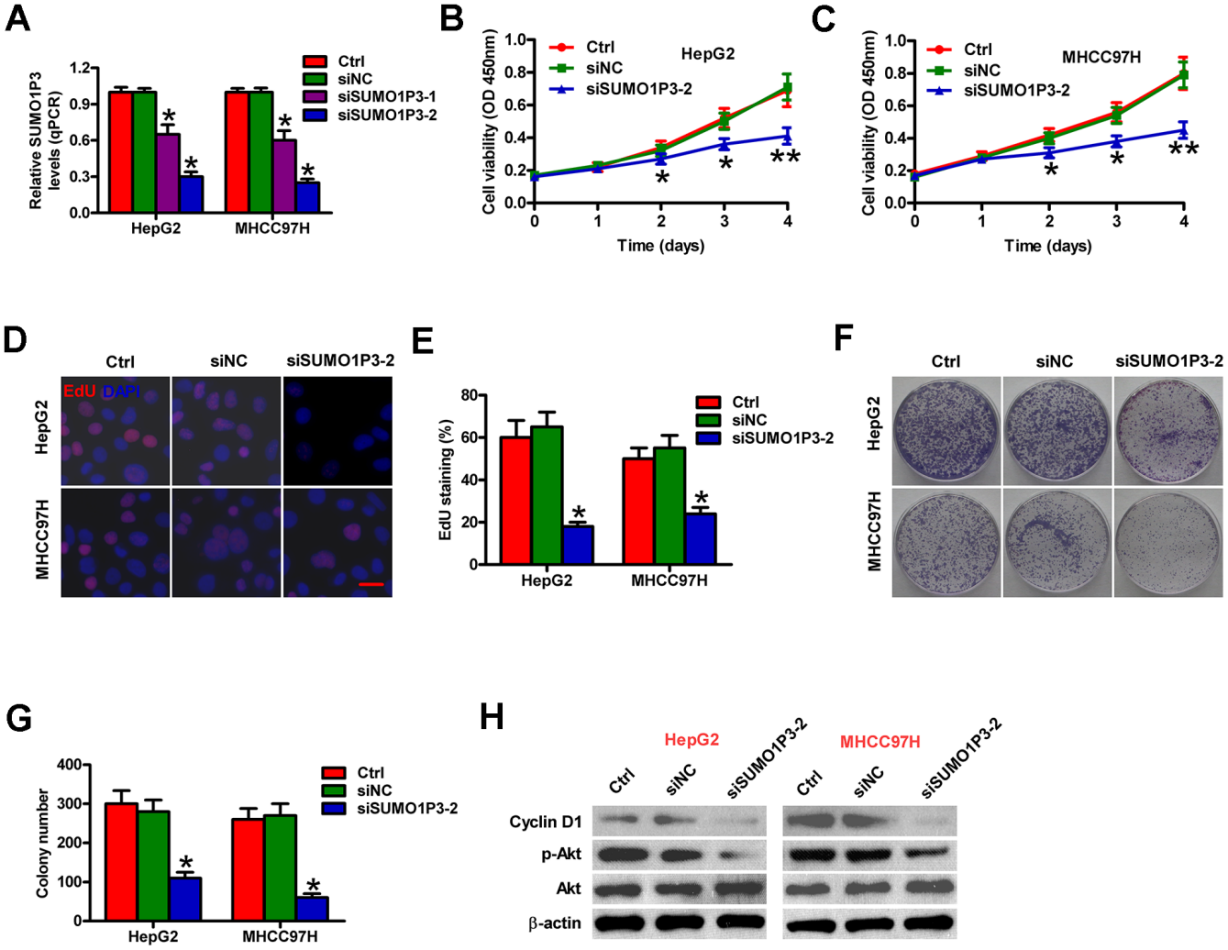
**Inhibitory effects of SUMO1P3 knockdown on HCC cell proliferation *in vitro*.** MHCC97H and HepG2 cells were not transfected (control) or transfected with siSUMO1P3 or siNC. (**A**) The expression of SUMO1P3 was detected by qPCR assays. GAPDH was used as the endogenous control. (**B**, **C**) Cell viability was measured using CCK8 assay at 1, 2, 3, and 4 days after transfection. (**D**) EdU staining was used to detect cell proliferation. Scale bar: 5 μm. (**E**) Percentage of EdU-positive staining in (**D**). (**F**) Representative photos of colony formation. (**G**) Colony number was calculated in (**F**). (**H**) Western blot was conducted to analyze the expression of cyclin D1, p-Akt, and Akt. β-actin was used as endogenous control. All data are represented as the mean ± SD of three replicates. *P < 0.05, **P < 0.01 vs. Ctrl or siNC group.

To determine the biological roles of SUMO1P3 in HCC cell proliferation, we used siRNA technology to knockdown SUMO1P3 expression in MHCC97H and HepG2 cells. Two sets of siRNA (siSUMO1P3-1 and siSUMO1P3-2) against SUMO1P3 were transfected into HCC cells. siSUMO1P3 transfection dramatically reduced SUMO1P3 level in MHCC97H and HepG2 cells compared to the siNC-transfected and control cells (Figure 3A). Because siSUMO1P3-2 has significantly higher silencing efficiency than siSUMO1P3-1, siSUMO1P3-2 is selected for all the subsequent experiments. SUMO1P3 knockdown reduced MHCC97H and HepG2 cell viability (Figure 3B, 3C). EdU incorporation experiment demonstrated that SUMO1P3 silencing inhibited MHCC97H and HepG2 cell proliferation (Figure 3D, 3E). Also, SUMO1P3 downregulation in MHCC97H and HepG2 cells lead to fewer and smaller colonies than siNC-transfected or control cells (Figure 3F, 3G). Mechanistically, SUMO1P3 depletion dramatically decreased cyclin D1 expression and Akt phosphorylation (Figure 3H). These data suggested that SUMO1P3 silencing decreases HCC cell proliferation *in vitro*.

### SUMO1P3 silencing promotes HCC cell apoptosis *in vitro*

**Figure 4:**
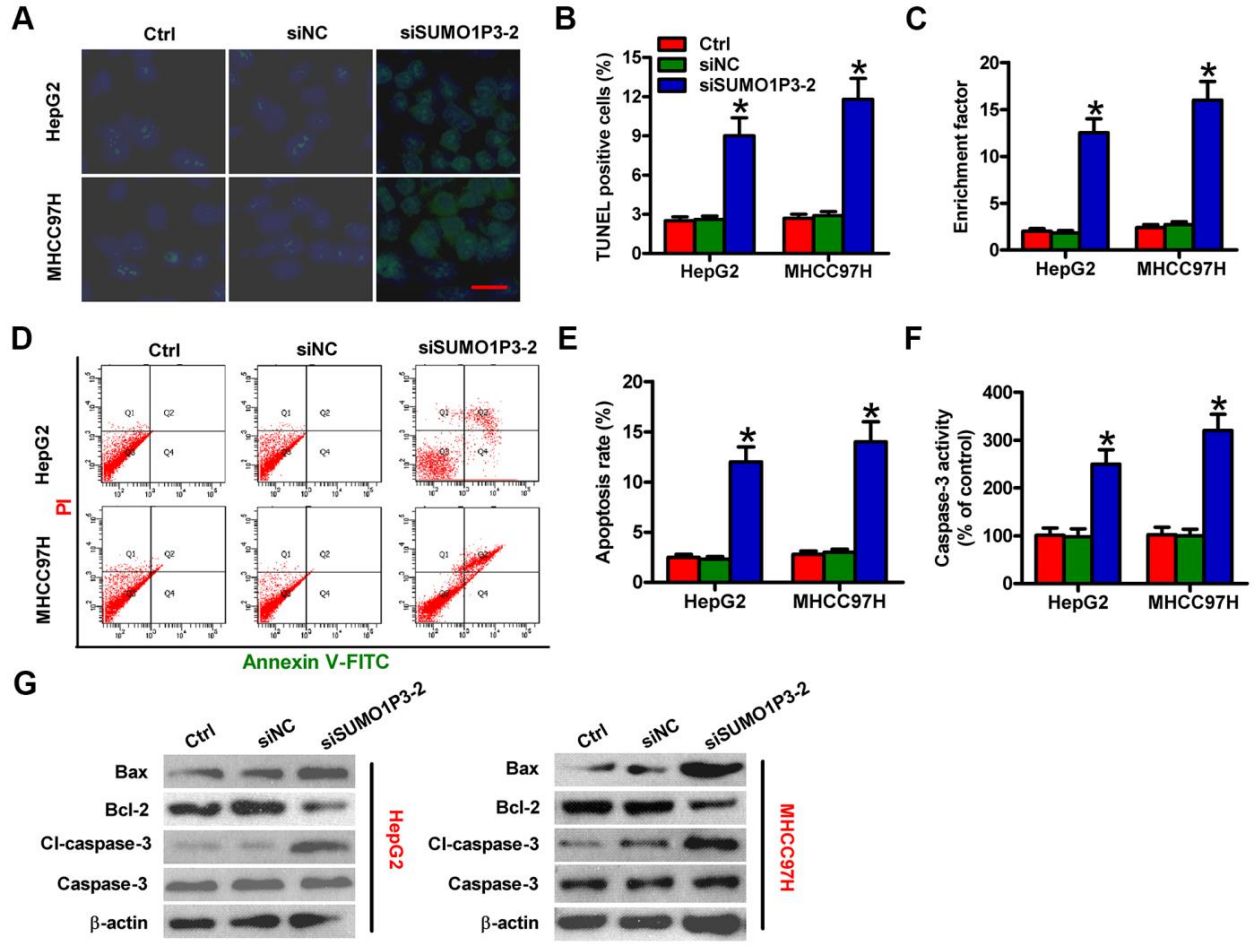
**SUMO1P3 silencing induced HCC cell apoptosis *in vitro*.** MHCC97H and HepG2 cells were not transfected (control) or transfected with siSUMO1P3-2 or siNC. Cell apoptosis was measured via TUNEL (**A**, **B**), DNA fragmentation (**C**) and flow cytometry (**D**) assays. Scale bar in (**A**): 10 μm. (**E**) The percentage of apoptotic cells in (**D**) was assessed. (**F**) Caspase-3 activity was detected by using a commercial kit. (**G**) The levels of Bax, Bcl-2, cl-caspase-3, and caspase-3 were analyzed via Western blot. β-actin was used as endogenous control. All data are represented as the mean ± SD of three replicates. *P < 0.05 vs. Ctrl or siNC group.

Next, the roles of SUMO1P3 were investigated in apoptosis of HCC cells. The results of TUNEL assay showed more apoptotic cells in siSUMO1P3-transfected MHCC97H and HepG2 cells than that in siNC-transfected or control cells (Figure 4A, 4B). DNA fragmentation assay showed notable increased apoptotic ratios of SUMO1P3-silenced MHCC97H and HepG2 cells (Figure 4C). Flow cytometry analysis demonstrated that SUMO1P3 knockdown markedly induced HCC cell apoptosis (Figure 4D, 4E). Moreover, caspase-3 activity was remarkably elevated in SUMO1P3-depleted MHCC97H and HepG2 cells (Figure 4F). To discover the underlying mechanisms on the contribution of SUMO1P3 to HCC cell apoptosis, Western blot was performed to determine the expression of Bax, Bcl-2, cl-caspase-3, and caspase-3. Depletion of SUMO1P3 resulted in the significant increase in Bax and cl-caspase-3 levels and decrease in Bcl-2 expression (Figure 4G). These findings indicated that SUMO1P3 knockdown increases HCC cell apoptosis *in vitro*.

### SUMO1P3 knockdown suppresses the migration and invasion of HCC cells *in vitro*

**Figure 5:**
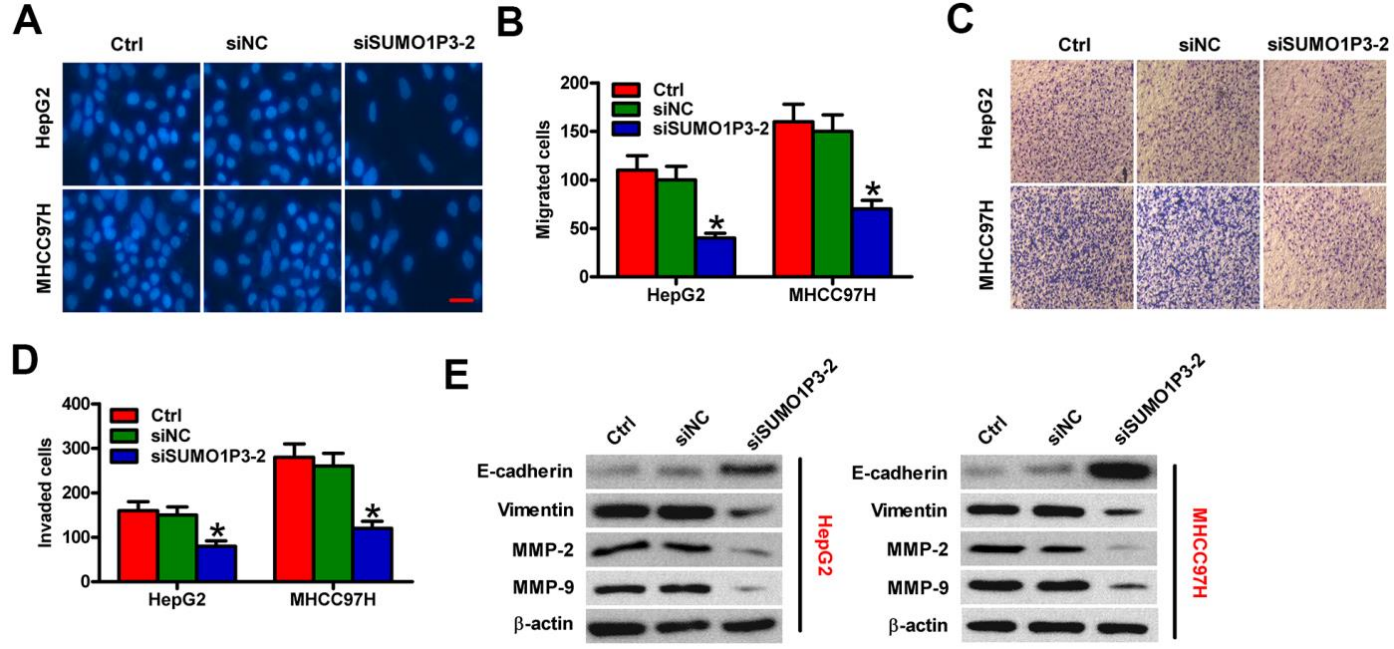
**SUMO1P3 knockdown inhibited HCC cell migration and invasion *in vitro*.** MHCC97H and HepG2 cells were untransfected (control) or transfected with siSUMO1P3 or siNC. Transwell assays were performed to evaluate the migration (**A**) and invasion (**C**) of MHCC97H and HepG2 cells. 40 × magnification in (**C**). The numbers of migrated (**B**) and invaded (**D**) cells were calculated. (**E**) The expression of E-cadherin, vimentin, MMP-2, and MMP-9 was analyzed via Western blot. β-actin was used as endogenous control. All data are represented as the mean ± SD of three replicates. *P < 0.05 vs. Ctrl or siNC group.

To assess the roles of SUMO1P3 in HCC cell motility, Transwell assays were performed. SUMO1P3 silencing dramatically inhibited the migration of MHCC97H and HepG2 cells compared to siNC-transfected or control cells (Figure 5A, 5B). Similarly, cell invasion was markedly suppressed by SUMO1P3 knockdown (Figure 5C, 5D). To explore the underlying mechanism of the SUMO1P3-mediated motility in HCC cells, the expressions of E-cadherin, vimentin, MMP-2, and MMP-9, which are recognized as key molecules for cancer metastasis, were detected by Western blot. As expected, SUMO1P3 knockdown significantly decreased the levels of vimentin, MMP-2, and MMP-9 and increased E-cadherin expression in HCC cells (Figure 5E). These results suggested that SUMO1P3 depletion hampers migration and invasion of HCC cells *in vitro*.

### SUMO1P3 depletion retards HCC growth and lung metastasis *in vivo*

**Figure 6:**
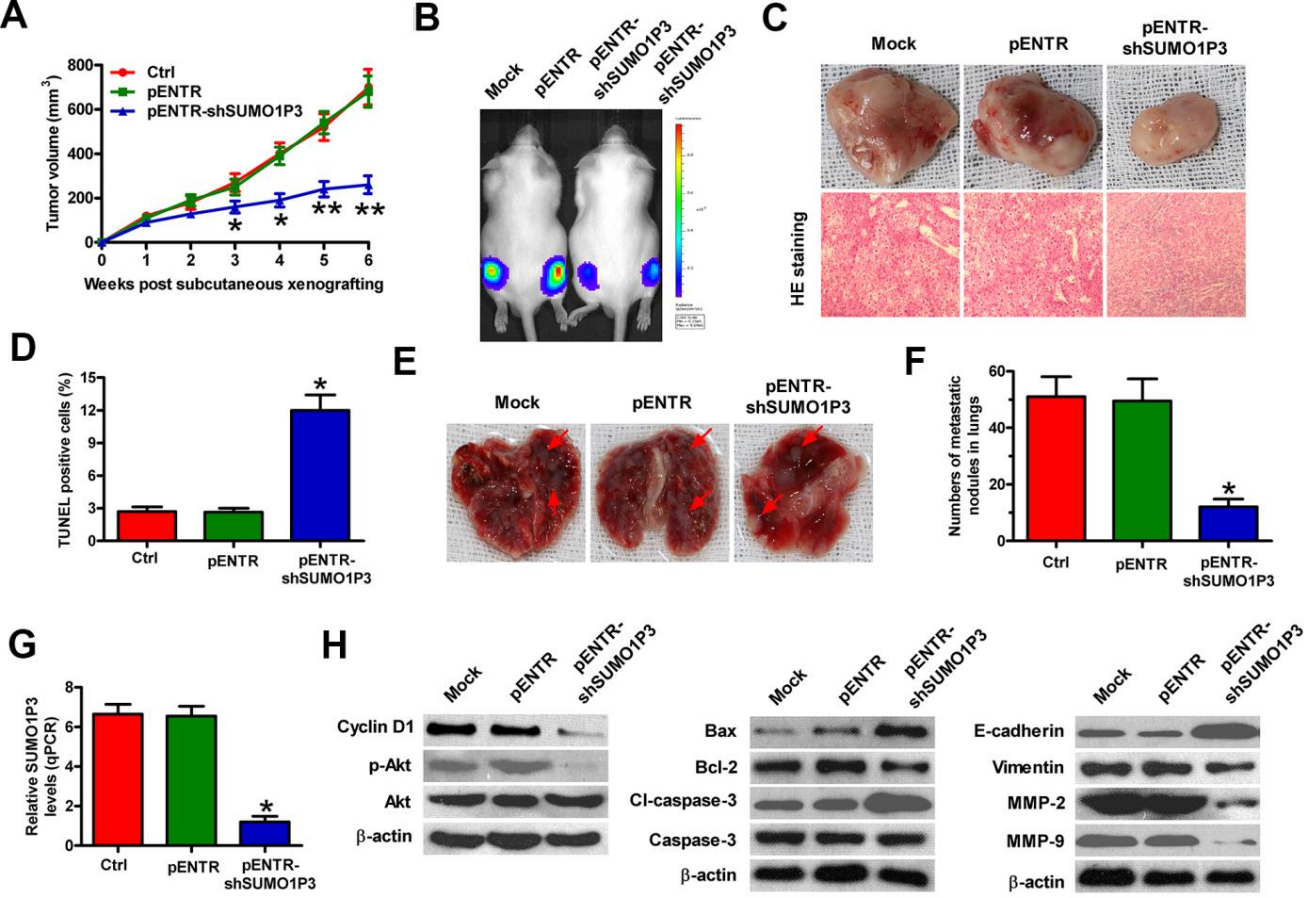
**SUMO1P3 depletion impeded HCC growth and metastasis *in vivo*.** Four-week-old male SCID mice were inoculated subcutaneously into the hind flanks or injected via tail vein with MHCC97H-luc cells with stably expressing shNC or shSUMO1P3-2. (**A**) Tumor volumes were monitored and calculated every week for 6 weeks. (**B**) Representative images of the mice (Mock and pENTR (n=10), or pENTR-shSUMO1P3 and pENTR-shSUMO1P3 (n=10)) photographed with an IVIS Imaging System at days 14 after inoculation. (**C**) Representative general photographs and HE staining assay of the harvested primary tumors at 6 weeks after subcutaneous xenografting. 100 × magnification in HE sections. (**D**) TUNEL assay was carried out to determine cell apoptosis in the tumor tissues of the xenografts. The percentage of TUNEL-positive cells was calculated. (**E**, **F**) At 8 weeks after injection through the tail vein, the lungs were harvested and photographed, and the numbers of pulmonary metastatic nodules was counted. (**G**) SUMO1P3 expression in tumor tissues was measured via qPCR assay. GAPDH was used as the endogenous control. (**H**) Representative results of Western blot analyses of cyclin D1, p-Akt, Akt, Bax, Bcl-2, cl-caspase-3, caspase-3, E-cadherin, vimentin, MMP-2, and MMP-9 in tumor tissues. β-actin was used as endogenous control. All data are represented as the mean ± SD of three replicates. *P < 0.05 vs. Mock or pENTR group.

To investigate the roles of SUMO1P3 in HCC growth and lung metastasis *in vivo*, heterotopic transplantation and metastatic models were established by subcutaneously or venously injecting with MHCC97H-luc cells stably expressing shSUMO1P3 or shNC. Compared with control or shNC group, SUMO1P3-depleted group exhibited significant reduction in tumor volume (Figure 6A). SUMO1P3-silenced mice showed slow tumor growth compared to the control or shNC-treated animals (Figure 6B, 6C). TUNEL-positive cells were much more in SUMO1P3-depleted tumors than those derived from control or shNC xenografts (Figure 6D). The metastatic nodules in the lungs were significantly reduced in the SUMO1P3-silenced group (Figure 6E, 6F). At the molecular level, SUMO1P3 depletion reduced SUMO1P3, cyclin D1, p-Akt, Bcl-2, cl-caspase-3, vimentin, MMP-2 levels, and MMP-9 but enhanced Bax and E-cadherin expression (Figure 6G, 6H). These results indicated that SUMO1P3 depletion hinders the growth and lung metastasis of HCC *in vivo*.

## DISCUSSION

In this study, several key findings are listed as follows. First, SUMO1P3 was significantly upregulated in HCC tissues and cells. Second, SUMO1P3 upregulation was closely correlated with tumor size and number, poor differentiation, lymphatic and distant metastasis, TNM stage, and poor outcomes of HCC patients. Third, SUMO1P3 overexpression promotes the proliferation, migration, and invasion and repressed serum starvation-caused apoptosis in normal liver cells. Fourth, SUMO1P3 silencing inhibited proliferation, migration, and invasion and promoted apoptosis of HCC cells *in vitro*. Fifth, SUMO1P3 knockdown hampered HCC growth and lung metastases *in vivo*. Collectively, these results suggested that SUMO1P3 plays critical roles in HCC progression by increasing cell viability, proliferation and invasion, and decreasing cell apoptosis, providing evidence that SUMO1P3 may be a useful prognostic marker and a prospective target for HCC therapy.

Globally, HCC, the most prevalent liver cancer type, is the main cause of cancer-related death owing to the high relapse and/or metastasis rate [3, 4]. Despite substantial efforts in HCC diagnostic and therapeutic strategies, the 5-year survival rate are still not improved significantly for HCC patients [16]. Thus, discovering the potential mechanisms in the development and recurrence of HCC and exploring biomarkers for HCC patients that are more effective than existing ones are meaningful for improving the bad outcomes. Previous literatures reported that SUMO1P3 is a potential biomarker in the diagnosis of gastric and bladder cancers and that SUMO1P3 promotes bladder cancer growth and metastasis [12, 13]. Nevertheless, the clinical importance of SUMO1P3 in HCC is yet to be fully investigated. In the current study, SUMO1P3 level was significantly higher in HCC tissues than the corresponding noncancerous samples, suggesting its clinical significance. High expressions of SUMO1P3 were positively correlated with some clinicopathological parameters, including tumor size and number, poor differentiation, lymphatic and distant metastases, and TNM stage. The above results suggest that SUMO1P3 may play oncogenic roles in HCC progression. The prognostic value of SUMO1P3 was confirmed to further evaluate its possible clinical applications. The patients with high SUMO1P3 expression showed higher 5-year survival rate than the SUMO1P3 low-expression patients. Thus, SUMO1P3 may serve as a potential biomarker for HCC prognosis.

LncRNAs are found to regulate gene at the epigenetic, transcriptional, post-transcriptional, and translational levels [17, 18]. Accumulating evidence has demonstrated that lncRNAs participate in tumorigenesis by controlling several pathways [6–9]. The aberrant expression of lncRNAs may contribute to tumor cell proliferation and ultimately lead to aggressive growth and metastasis of tumors [19, 20]. Several lncRNAs have been reported to affect HCC malignant behaviors [21–24]. For instance, lncRNA CCHE1 knockdown significantly inhibits cell proliferation and promotes apoptosis of HCC cells by inactivating the ERK/MAPK pathway [21]. SNHG20 promotes the epithelial–mesenchymal transition (EMT) process to facilitate HCC cell invasion [22]. Meanwhile, CCAT2 induces migration and invasion of HCC cells *in vitro* and enhances EMT by regulating vimentin, E-cadherin, and Snail2 expression [23]. UC001kfo promotes proliferation and motility of HCC cells *in vitro* and enhances HCC growth and metastasis ability *in vivo* [24]. Reportedly, SUMO1P3 promotes the proliferation, migration, invasion, and apoptosis resistance of bladder cancer cells [13]. However, as far as we know, the biological roles and underlying mechanisms of SUMO1P3 have not been previously investigated in HCC. In this study, we demonstrated that SUMO1P3 promotes proliferation, migration and invasion of HCC cells and suppresses HCC cell apoptosis *in vitro* and accelerates HCC growth and metastasis *in vivo*. These observations indicated that SUMO1P3 serves as an oncogene to facilitate HCC progression and metastasis.

Uncontrolled cell proliferation and apoptosis resistance are the well-known characteristics of HCC. cyclin D1, mediating G1 to S phase transition, is a well-established oncogene and important for the development and progression of several cancers [25]. Cyclin D1 is upregulated in HCC, and cyclin D1 knockdown attenuates the proliferative capability of HCC cells [26]. Akt contributes to cancer cell survival and Akt/cyclin D1 signaling appears to promote HCC growth [27]. Meanwhile, Akt signaling can inhibit HCC cell apoptosis [28]. Akt helps accessory proteins to bind to transcript factor to regulate anti-apoptotic gene expression, such as Bcl-2 [29]. Bcl-2 is a common cell death suppressor, and Bax is a pro-apoptotic protein that interacts with Bcl-2. The equilibrium between pro- and anti-apoptotic proteins regulates cell apoptosis by facilitating the permeability of mitochondrial outer membranes. This permeability leads to cytochrome c entry into the cytoplasm and caspase-3 activation [30]. High Bcl-2 and low Bax levels are observed in HCC cells, and a decreased ratio of Bcl-2/Bax increases HCC cell apoptosis [31]. In the current study, SUMO1P3 silencing reduced the levels of pro-survival proteins (cyclin D1, p-Akt, and Bcl-2) and increased the pro-apoptotic molecules (Bax and cl-caspase-3) expression *in vitro* and *in vivo*. These data suggested that the SUMO1P3-increased HCC growth may depend on Akt-steered signaling.

Distal metastasis is a main reason for HCC-related mortality. The dissemination of tumor cells from the primary site to the target organs is a complicated process that involves the interaction of diverse molecules to control cell motility and invasiveness [32]. EMT is a typical feature in HCC with metastasis [33]. In the process of EMT, the epithelial cells loss its epithelial markers, such as E-cadherin, and gain the mesenchymal markers, such as vimentin [34]. In this study, SUMO1P3 silencing leads to upregulation of E-cadherin and downregulation of vimentin in HCC cells *in vitro* and *in vivo*. MMPs result in the basement membrane and the extracellular matrix degradation which are necessary for tumor cell motility [35]. Upregulation of MMP-2 and MMP-9 are positively associated with HCC metastasis [36]. We here demonstrated that SUMO1P3 depletion reduced expressions of MMP-2 and MMP-9 in HCC cells *in vitro* and *in vivo*. These findings suggested that SUMO1P3 promotes HCC metastasis by facilitating EMT and extracellular matrix degradation.

This study has some limitations. We didn’t investigate how SUMO1P3 promoted the down/upregulation of the analyzed proteins (Cyclin D1, p-Akt, E-cadherin, vimentin, etc). The speculations that SUMO1P3 possibly regulates a series of miRNAs, and the regulated miRNAs directly targets Cyclin D1, p-Akt, E-cadherin, and vimentin or targets their upstream molecules, need to be verified in the future.

In conclusion, upregulation of SUMO1P3 was observed in HCC tissues and cells. High SUMO1P3 expression was positively associated with tumor growth and metastasis and predicted poor outcomes in HCC patients. SUMO1P3 knockdown significantly suppressed the proliferation, migration, invasion and apoptosis resistance in HCC cells *in vitro*, whereas SUMO1P3 overexpression in normal liver cells exhibited the opposite effects. Consistently, the results of animal experiments showed that SUMO1P3 depletion hindered HCC growth and lung metastasis. Overall, these observations indicated the potential of SUMO1P3 for targeted therapy in HCC patients.

## MATERIALS AND METHODS

### Patients and clinical samples

HCC and the corresponding adjacent noncancerous samples were collected from 103 patients without prior chemotherapy or radiotherapy, who underwent surgical resection in the First Affiliated Hospital of Henan University of Science and Technology (Luoyang, China) between July 2009 and June 2011. All samples were harvested instantly following resection and stored in liquid nitrogen until analysis. Clinical staging was performed by experienced pathologists in accordance with the recommendations from American Joint Committee on Cancer. Follow-up data were obtained primarily through outpatient examination or telephone. The clinical data of enrolled patients are summarized in Table 1. Written informed consent was signed by every participant. This study was approved by the Internal Review and Ethics Boards of the First Affiliated Hospital of Henan University of Science.

### Cell culture

Six human HCC cell lines (BEL-7402, MHCC97L, Hep3B, Huh7, MHCC97H, and HepG2), normal hepatocyte cells LO2, and HEK293T cell line were purchased from the ATCC (Manassas, VA, USA). The cells were confirmed by the provider using gene profiling analysis. All cells were used within 6 months. All cells were cultivated in DMEM (Gibco, BRL, Carlsbad, CA, USA) containing 10% fetal bovine serum (FBS; Gibco), 100 U/mL penicillin, and 100 mg/mL streptomycin (Sigma–Aldrich, St. Louis, MO, USA) and incubated at 37° C and 5% CO_2_ in a humidified atmosphere. The 2–4 passage cells grown to 80% of confluence were used for experiments.

### Lentivirus construction

For lentiviral construction, the sequence of short hairpin RNA (shRNA) targeting SUMO1P3 (shSUMO1P3) or a scrambled shRNA (shNC) was cloned into pENTR vector (Addgene, Cambridge, MA, USA). pMDLg/pRRE and pRSV-REV are lentiviral packaging plasmids. HEK293T cells were cotransfected with the recombinant pENTR, pMDLg/pRRE and pRSV-REV plasmids. After 48 h, the medium was collected for the purification of viruses. MHCC97H-luciferase (MHCC97H-luc) cells were incubated with virus-containing supernatants in the presence of 8 mg/ml polybrene. The stable-infected cells were screened for 2 weeks by 5 μg/mL of puromycin (Sigma).

### Cell transfection

The pcDNA3.1-SUMO1P3 plasmid was provided by InvivoGen (Hong Kong, China). The negative control (siNC) and siRNA targeting SUMO1P3 (siSUMO1P3) were obtained from GenePharma (Shanghai, China). The siSUMO1P3-1 and siSUMO1P3-2 sequences were 5′-TGTGGTAGCGGAAGTTACTGCAGCT-3′ and 5′-TGGCCCTGATGTTCTAGCATGTGAT-3′, respectively. Cell transfection was performed by using Lipofectamine 2000 reagent (Invitrogen, Carlsbad, CA, USA) in accordance with the manufacturer’s protocol. LO2 cells were transfected with 3 μg of pcDNA3.1-SUMO1P3 or pcDNA3.1 plasmids and MHCC97H and HepG2 cells were transfected with 100 nM siSUMO1P3-1 or siSUMO1P3-2 or siNC.

### RNA extraction and quantitative real-time PCR (qPCR)

TRIzol reagent (Invitrogen) was used to extract the total RNA from the samples and the cultured cells in accordance with the manufacturer’s protocols. Complementary DNA was reversely transcribed from RNA by using the SuperScript™ II Reverse Transcriptase kit (Invitrogen). The qPCR actions were performed with the SYBR Premix Ex Taq™ (TaKaRa, Otsu, Shiga, Japan) on an ABI 7500 Real-Time PCR system (Applied Biosystems, Foster City, USA). The primers used for qPCR were listed below: for SUMO1P3, 5′-ACTGGGAATGGAGGAAGA-3′ (forward) and 5′-TGAGAAAGGAT TGAGGGAAAAG-3′ (reverse); for GAPDH, 5′-GACTCATGACCACGTCCATGC-3′ (forward) and 5′-AGAGGCAGGGATGATG TTCTG-3′ (reverse). Glyceraldehyde-3-phosphate dehydrogenase (GAPDH) was used as the normal control. The relative expression of SUMO1P3 was calculated using the 2^−ΔΔCt^ method.

### Cell viability assay

Cell Counting Kit-8 (CCK-8; Beyotime, Shanghai, China) was used to evaluate cell viable ability. MHCC97H and HepG2 cells were seeded into 96-well plates in triplicate at 1,000 cells per well prior to transfection with siSUMO1P3 or siNC. At 1, 2, 3, or 4 days after transfection, the cells were added with 10 μL of CCK-8 (5 mg/mL) according to the manufacturer’s instruction. Following incubation for 2 h at 37° C, optical density (OD) at 450 nm was measured by a microplate reader (Bio-Rad, Hercules, CA, USA).

### 5-Ethynyl-2′-deoxyuridine (EdU) incorporation assay

The EdU Apollo DNA *in vitro* kit (RiboBio, Guangzhou, China) was employed to assess the influence of SUMO1P3 on cell proliferation. MHCC97H, HepG2 cells and LO2 cells were seeded into 96-well plates at a density of 1 × 10^3^ cells per well. After transfection with pcDNA3.1-SUMO1P3 or pcDNA3.1 plasmids (for MHCC97H and HepG2 cells) and siSUMO1P3 or siNC mimics (for LO2 cells) for 48 h, 50 μM of EdU (100 μL/well) was added to the cells and incubated at 37° C for 2 h. Then, the cells were fixed with 4% paraformaldehyde for 20 min at room temperature and washed with PBS thrice. Following incubation with 50 μL of glycine (2 mg/mL) for 5 min, Apollo solution was added to the cells and incubated for 30 min in the dark at room temperature. Subsequently, the cells were permeabilized with 0.5% Triton X and stained with DAPI (100 μL) for 30 min in the dark at room temperature. After washing by PBS thrice, five random fields were selected for visualizing and photographing the EdU-positive cells using the fluorescent microscopy (Carl-Zeiss, Berlin, Germany). EdU-positive cells were counted using “Manually Count Objects” dropin of MetaMorph 6.2 image processing software (Molecular Devices). The percentage of EdU-positive cells to the total cells (DAPI-positive cells) was calculated to evaluate the proliferative ability.

### Colony formation assay

For colony formation assay, siSUMO1P3- or siNC-transfected MHCC97H and HepG2 were seeded into six-well plates at 1,000 cells per well and grown in complete medium for 2 weeks. The medium was freshly changed every 3 days. After fixing with 4% paraformaldehyde, the cells were stained with 0.5% crystal violet (Sigma). The panoramic view of each well of six-well plate was obtained by a camera. For quantifying crystal violet-staining colony formation of HCC cells in wells, five fields of crystal violet-stained colonies were randomly selected to visualize and photograph using the microscopy (Carl-Zeiss) and automatic cell counting was conducted in MetaMorph Software (Molecular Devices).

### DNA fragmentation assay

Apoptotic cell death was measured by detecting cytoplasmic histone-associated-DNA-fragments using a Cell Death Detection ELISA Plus Kit (Roche Applied Science, Mannheim, Germany) following the manufacturer’s protocol. Briefly, siSUMO1P3- or siNC-transfected MHCC97H and HepG2 cells were lyzed by RIPA lysis buffer (Beyotime) for 30 min at room temperature and centrifuged at 200 ×g for 10 min. The supernatant (20 μL) was transferred onto the streptavidin-coated plate followed by 80 μL of freshly-prepared peroxidase conjugated anti-DNA and biotin-labeled antihistone incubation for 2 h at room temperature. After washing with incubation buffer, the substrate solution (2,2′-azino-di-[3-ethylbenzthiazolinesulfonate] diammonium salt; 100 μL) was added and incubated for 20 min. The absorbance was measured by a microplate reader (Bio-Rad) at 405 nm (reference wavelength at 490 nm).

### Flow cytometry assay for apoptosis

Annexin V-fluorescein isothiocyanate (FITC) Apoptosis Detection Kit (BD Bioscience, San Jose, CA, USA) was purchased to measure cell apoptosis. MHCC97H and HepG2 cells were harvested after transfection with siSUMO1P3 or siNC for 48 h. The cells were then centrifuged at 1,000 ×g for 5 min and washed with PBS thrice. A total of 5 × 10^5^ transfected cells were collected and 500 μL binding buffer was added to the cells for resuspension. Annexin V-FITC (10 μL) was added for incubation at 37° C and light-proof for 15 min, followed by 5 μL of propidium iodide (PI) added in the mixture for reaction in the dark at 37° C for 30 min. Cell apoptosis was determined by BD FACSCalibur flow cytometry (BD Bioscience) and analyzed by the CellQuest software (BD Bioscience).

### Caspase-3 activity assay

Caspase-3 Colorimetric Assay kit (Abcam, Cambridge, UK) was obtained to assess caspase-3 activity as per the protocol of the manufacturer. 24 h-serum-starved LO2 cells (1 × 10^6^) were transfected with pcDNA3.1 or pcDNA3.1-SUMO1P3 plasmids or MHCC97H and HepG2 cells (1 × 10^6^) were transfected with siNC or siSUMO1P3. At 48 h after transfection, all the cells were lysed by lysis buffer on ice for 30 min and centrifuged at 1,000 ×g for 1 min. The supernatants were harvested for protein measurement using a BCA assay kit (Beyotime). Approximately 50 μL samples (150 μg protein) were incubated with 2 × reaction buffer (50 μL) containing the substrate (N-acetyl-Asp-Glu-Val-Asp-pNA; 200 μM final concentration) for 2 h at room temperature. Caspase-3 activity was determined by measuring the absorbance at 405 nm.

### Terminal transferase-mediated dUTP nick end-labeling (TUNEL) assay

Apoptotic cells were detected by TUNEL staining assay using the *In Situ* Cell Death Detection kit (Roche, Mannheim, BW, Germany) in accordance with the protocols of manufacturers. For cells, the untransfected and siSUMO1P3- or siNC-transfected MHCC97H and HepG2 cells were seeded on the glass slides and incubated for 24 h at 37° C. Following fixing in 80% glycerol at room temperature for 1 h, the cells were washed with PBS (pH 7.4) and incubated in permeabilization solution (2% Triton X-100) for 2 min at 4° C. Subsequently, the cells were incubated with FITC-labeled terminal deoxynucleotidyl transferase (TdT) nucleotide mix (Promega, Madison, WI, USA) for 1 h at 37° C. FITC-labeled TdT was not provided to the negative control. After washing with PBS twice, the slides were incubated with 10 mg/mL of 4′,6-diamidino-2-phenylindole (DAPI; Sigma). For tissues, the samples were fixed in 10% formalin overnight followed by embedding in paraffin. Then, the samples were nonserially cut into 4-μm thickness and mounted on polylysine-coated slides. After deparaffinization with xylene and rehydration with ethanol, the sections were washed with PBS and FITC-labeled TdT was added to the slides for incubation at 37° C for 1 h. Subsequently, the sections were washed with PBS twice following with 10 mg/mL of DAPI incubation. The stained sections were visualized and photographed under an inverted fluorescent microscope (Carl-Zeiss). The apoptotic cells were expressed as the percentage of TUNEL-positive cells to the total cells (DAPI-stained cells).

### Transwell assays

Transwell assays were performed to evaluate cell migration and invasion using the transwell inserts (8-μm pore size; Corning Costar, NY, USA). At 48 h after LO2 cells were transfected with pcDNA3.1-SUMO1P3 or pcDNA3.1 plasmids or MHCC97H and HepG2 cells were transfected with siSUMO1P3 or siNC mimics, 5 × 10^4^ cells were placed in the upper chamber of the inserts pre-coated with (for invasion) or without (for migration) Matrigel. DMEM (600 μL) containing 10% FBS as a chemoattractant was added to the lower chamber and the cells were allowed to mobilize for 24 h. The cells that migrated or invaded the membranes were fixed with methanol for 30 min, followed by staining with DAPI or crystal violent for another 30 min. The cells on the lowed surface of the membranes were counted by fluorescent microscopy (Carl-Zeiss). Each condition was performed in three chambers and the average number of cells in triplicates was determined for each group.

### Western blot analysis

The fresh tissues were lyzed with RIPA buffer (Beyotime) for protein extraction. Then, the samples were centrifuged and the supernatants were collected. Protein concentrations were determined by a BCA assay kit (Beyotime). Equal amount of proteins (30 μg) were loaded onto a 10% SDS-PAGE gel, followed by transferring onto nitrocellulose membranes (Millipore, Bedford, MA, USA). After blocking with 5% skimmed milk at room temperature for 2 h, the membranes were incubated with several primary antibodies, including cyclin D1, Akt, and phosphorylated (p)-Akt (Ser473) (all from Cell Signaling Technology, Danvers, MA, USA), Bax, Bcl-2, caspase-3, and cleaved (cl)-caspase-3 (all from Abnova, Taiwan, China), Vimentin, E-cadherin, matrix metalloproteinase (MMP)-2, MMP-9, and β-actin (all from Abcam) overnight at 4° C. Subsequently, secondary antibodies (Sigma) conjugated with horseradish peroxidase were added to the membranes and incubated at 37° C for 1 h. The immunoblots were detected by an enhanced chemiluminescence kit (Santa Cruz, Dallas, TX, USA).

### Subcutaneous xenograft and metastasis mouse models

pLV-luc (Inovogen Biotechnology, Delhi, India) was procured for infecting MHCC97H cells to establish a luciferase-expressing cell line. Briefly, the infected MHCC97H cells were seeded in 24-well plates and 200 μg/mL of puromycin was added to each well for selection of the cells with stably-expressed luciferase. Sixteen days later, a single clone was selected and designated as the MHCC97H-luc cell line.

Twenty four male SCID mice (4-week-old, weighing 18–22 g) were obtained from the Institute of Zoology, Chinese Academy of Sciences (Beijing, China) and maintained under specific pathogen-free conditions. The mice were housed at 22° C to 24° C and light/dark cycle of 12 h/12 h with free access to water and food. Animal experiments were performed following the guidelines from the Care and Use of Laboratory Animals of the National Institutes of Health. This study was approved by the Committee on the Ethics of Animal Experiments of the First Affiliated Hospital of Henan University of Science.

For *in vivo* tumor growth assay, MHCC97H-luc cells (1 × 10^6^) stably expressing shNC or shSUMO1P3 were subcutaneously injected into the hind flank of the SCID mice (n = 6 mice/group). Tumor size was measured once a week by a caliper and tumor volume was estimated according to the formula: tumor volume = length × width^2^/2. Two weeks later, D-luciferin (Promega, Madison, WI, USA) were intraperitoneally injected into the mice (150 mg/kg per mouse). Afterwards, the mice were anesthetized under isoflurane. Bioluminescence imaging of the tumor-bearing mice was captured by the Xenogen IVIS imaging system (Xenogen Corp., Berkeley, CA, USA) at 15 min after D-luciferin injection. Regions of interest from displayed images were identified and quantified as photons per second per centimeter squared per steradian (p/s/cm^2^/sr) using Living Image software (Xenogen Corp.). At six weeks after tumor cell implantation, the mice were euthanized under anesthetic and tumors were dissected for picture acquisition. Partial tumor tissues were immediately fixed and embedded with paraffin for hematoxylin and eosin (HE) staining and TUNEL assay and the remaining samples were frozen at −80° C for qPCR and Western blot analyses.

For *in vivo* metastasis assay, MHCC97H-luc cells (5 × 10^5^) stably expressing shNC or shSUMO1P3 were injected into the tail vein of the SCID mice (n = 6 mice/group). The general health condition of the mice was monitored and the primary tumor- or metastasis-related morbidity was evidenced. At eight weeks post-inoculation, the mouse lungs were collected and photographed, and metastatic nodules in the lungs were counted.

### Statistical analysis

SPSS software (version 19.0; SPSS Inc., Chicago, IL, USA) was adopted for statistical analyses. Data were expressed as mean ± standard deviation (SD). Student’s t-test was employed to compare differences between two groups and one-way ANOVA test was utilized to analyze significances among three groups or more. Chi-squared test was used to evaluate the qualitative data. The survival curve was plotted by the Kaplan–Meier method and the log-rank test was used to determine the survival difference. Statistical significance was considered at *P* < 0.05.

## References

[1] Llovet JM, Burroughs A, Bruix J. Hepatocellular carcinoma. Lancet. 2003; 362:1907–17. 10.1016/S0140-6736(03)14964-1 14667750

[2] Chen W, Zheng R, Baade PD, Zhang S, Zeng H, Bray F, Jemal A, Yu XQ, He J. Cancer statistics in China, 2015. CA Cancer J Clin. 2016; 66:115–32. 10.3322/caac.21338 26808342

[3] El-Serag HB, Rudolph KL. Hepatocellular carcinoma: epidemiology and molecular carcinogenesis. Gastroenterology. 2007; 132:2557–76. 10.1053/j.gastro.2007.04.061 17570226

[4] Llovet JM. Liver cancer: time to evolve trial design after everolimus failure. Nat Rev Clin Oncol. 2014; 11:506–07. 10.1038/nrclinonc.2014.136 25091613 PMC12261308

[5] Hanazaki K, Kajikawa S, Shimozawa N, Mihara M, Shimada K, Hiraguri M, Koide N, Adachi W, Amano J. Survival and recurrence after hepatic resection of 386 consecutive patients with hepatocellular carcinoma. J Am Coll Surg. 2000; 191:381–88. 10.1016/S1072-7515(00)00700-6 11030243

[6] Ponting CP, Oliver PL, Reik W. Evolution and functions of long noncoding RNAs. Cell. 2009; 136:629–41. 10.1016/j.cell.2009.02.006 19239885

[7] Fatica A, Bozzoni I. Long non-coding RNAs: new players in cell differentiation and development. Nat Rev Genet. 2014; 15:7–21. 10.1038/nrg3606 24296535

[8] Mitra SA, Mitra AP, Triche TJ. A central role for long non-coding RNA in cancer. Front Genet. 2012; 3:17. 10.3389/fgene.2012.00017 22363342 PMC3279698

[9] Mercer TR, Dinger ME, Mattick JS. Long non-coding RNAs: insights into functions. Nat Rev Genet. 2009; 10:155–59. 10.1038/nrg2521 19188922

[10] Gutschner T, Hämmerle M, Eissmann M, Hsu J, Kim Y, Hung G, Revenko A, Arun G, Stentrup M, Gross M, Zörnig M, MacLeod AR, Spector DL, Diederichs S. The noncoding RNA MALAT1 is a critical regulator of the metastasis phenotype of lung cancer cells. Cancer Res. 2013; 73:1180–89. 10.1158/0008-5472.CAN-12-2850 23243023 PMC3589741

[11] Gupta RA, Shah N, Wang KC, Kim J, Horlings HM, Wong DJ, Tsai MC, Hung T, Argani P, Rinn JL, Wang Y, Brzoska P, Kong B, et al. Long non-coding RNA HOTAIR reprograms chromatin state to promote cancer metastasis. Nature. 2010; 464:1071–76. 10.1038/nature08975 20393566 PMC3049919

[12] Mei D, Song H, Wang K, Lou Y, Sun W, Liu Z, Ding X, Guo J. Up-regulation of SUMO1 pseudogene 3 (SUMO1P3) in gastric cancer and its clinical association. Med Oncol. 2013; 30:709. 10.1007/s12032-013-0709-2 23996296

[13] Zhan Y, Liu Y, Wang C, Lin J, Chen M, Chen X, Zhuang C, Liu L, Xu W, Zhou Q, Sun X, Zhang Q, Zhao G, Huang W. Increased expression of SUMO1P3 predicts poor prognosis and promotes tumor growth and metastasis in bladder cancer. Oncotarget. 2016; 7:16038–48. 10.18632/oncotarget.6946 26799188 PMC4941296

[14] Wang Y, Hu Y, Wu G, Yang Y, Tang Y, Zhang W, Wang K, Liu Y, Wang X, Li T. Long noncoding RNA PCAT-14 induces proliferation and invasion by hepatocellular carcinoma cells by inducing methylation of miR-372. Oncotarget. 2017; 8:34429–41. 10.18632/oncotarget.16260 28415780 PMC5470980

[15] Evans JR, Feng FY, Chinnaiyan AM. The bright side of dark matter: lncRNAs in cancer. J Clin Invest. 2016; 126:2775–82. 10.1172/JCI84421 27479746 PMC4966302

[16] Altekruse SF, McGlynn KA, Dickie LA, Kleiner DE. Hepatocellular carcinoma confirmation, treatment, and survival in surveillance, epidemiology, and end results registries, 1992-2008. Hepatology. 2012; 55:476–82. 10.1002/hep.24710 21953588 PMC3868012

[17] Qiu MT, Hu JW, Yin R, Xu L. Long noncoding RNA: an emerging paradigm of cancer research. Tumour Biol. 2013; 34:613–20. 10.1007/s13277-013-0658-6 23359273

[18] Eades G, Zhang YS, Li QL, Xia JX, Yao Y, Zhou Q. Long non-coding RNAs in stem cells and cancer. World J Clin Oncol. 2014; 5:134–41. 10.5306/wjco.v5.i2.134 24829860 PMC4014785

[19] Deng R, Liu B, Wang Y, Yan F, Hu S, Wang H, Wang T, Li B, Deng X, Xiang S, Yang Y, Zhang J. High Expression of the Newly Found Long Noncoding RNA Z38 Promotes Cell Proliferation and Oncogenic Activity in Breast Cancer. J Cancer. 2016; 7:576–86. 10.7150/jca.13117 27053956 PMC4820734

[20] Deng W, Wang J, Zhang J, Cai J, Bai Z, Zhang Z. TET2 regulates LncRNA-ANRIL expression and inhibits the growth of human gastric cancer cells. IUBMB Life. 2016; 68:355–64. 10.1002/iub.1490 27027260

[21] Peng W, Fan H. Long noncoding RNA CCHE1 indicates a poor prognosis of hepatocellular carcinoma and promotes carcinogenesis via activation of the ERK/MAPK pathway. Biomed Pharmacother. 2016; 83:450–55. 10.1016/j.biopha.2016.06.056 27427851

[22] Liu J, Lu C, Xiao M, Jiang F, Qu L, Ni R. Long non-coding RNA SNHG20 predicts a poor prognosis for HCC and promotes cell invasion by regulating the epithelial-to-mesenchymal transition. Biomed Pharmacother. 2017; 89:857–63. 10.1016/j.biopha.2017.01.011 28282787

[23] Xu Y, Wang B, Zhang F, Wang A, Du X, Hu P, Zhu Y, Fang Z. Long non-coding RNA CCAT2 is associated with poor prognosis in hepatocellular carcinoma and promotes tumor metastasis by regulating Snail2-mediated epithelial-mesenchymal transition. Onco Targets Ther. 2017; 10:1191–98. 10.2147/OTT.S127100 28280353 PMC5338976

[24] Pan Y, Qin T, Yin S, Zhang X, Gao X, Mu L. Long non-coding RNA UC001kfo promotes hepatocellular carcinoma proliferation and metastasis by targeting α-SMA. Biomed Pharmacother. 2017; 87:669–77. 10.1016/j.biopha.2017.01.018 28088733

[25] Musgrove EA, Caldon CE, Barraclough J, Stone A, Sutherland RL. Cyclin D as a therapeutic target in cancer. Nat Rev Cancer. 2011; 11:558–72. 10.1038/nrc3090 21734724

[26] Yu H, Jiang HL, Xu D, Jin JZ, Zhao ZM, Ma YD, Liang J. Transcription Factor MafB Promotes Hepatocellular Carcinoma Cell Proliferation through Up-Regulation of Cyclin D1. Cell Physiol Biochem. 2016; 39:700–08. 10.1159/000445661 27448450

[27] Ye X, Guo Y, Zhang Q, Chen W, Hua X, Liu W, Yang Y, Chen G. βKlotho suppresses tumor growth in hepatocellular carcinoma by regulating Akt/GSK-3β/cyclin D1 signaling pathway. PLoS One. 2013; 8:e55615. 10.1371/journal.pone.0055615 23383245 PMC3559476

[28] Chen TA, Wang JL, Hung SW, Chu CL, Cheng YC, Liang SM. Recombinant VP1, an Akt inhibitor, suppresses progression of hepatocellular carcinoma by inducing apoptosis and modulation of CCL2 production. PLoS One. 2011; 6:e23317. 10.1371/journal.pone.0023317 21826248 PMC3149645

[29] Hussain AR, Ahmed SO, Ahmed M, Khan OS, Al Abdulmohsen S, Platanias LC, Al-Kuraya KS, Uddin S. Cross-talk between NFkB and the PI3-kinase/AKT pathway can be targeted in primary effusion lymphoma (PEL) cell lines for efficient apoptosis. PLoS One. 2012; 7:e39945. 10.1371/journal.pone.0039945 22768179 PMC3386924

[30] Shamas-Din A, Kale J, Leber B, Andrews DW. Mechanisms of action of Bcl-2 family proteins. Cold Spring Harb Perspect Biol. 2013; 5:a008714. 10.1101/cshperspect.a008714 23545417 PMC3683897

[31] Yang F, Shi L, Liang T, Ji L, Zhang G, Shen Y, Zhu F, Xu L. Anti-tumor effect of evodiamine by inducing Akt-mediated apoptosis in hepatocellular carcinoma. Biochem Biophys Res Commun. 2017; 485:54–61. 10.1016/j.bbrc.2017.02.017 28189683

[32] Chaffer CL, Weinberg RA. A perspective on cancer cell metastasis. Science. 2011; 331:1559–64. 10.1126/science.1203543 21436443

[33] van Zijl F, Zulehner G, Petz M, Schneller D, Kornauth C, Hau M, Machat G, Grubinger M, Huber H, Mikulits W. Epithelial-mesenchymal transition in hepatocellular carcinoma. Future Oncol. 2009; 5:1169–79. 10.2217/fon.09.91 19852728 PMC2963061

[34] Österreicher CH, Penz-Österreicher M, Grivennikov SI, Guma M, Koltsova EK, Datz C, Sasik R, Hardiman G, Karin M, Brenner DA. Fibroblast-specific protein 1 identifies an inflammatory subpopulation of macrophages in the liver. Proc Natl Acad Sci USA. 2011; 108:308–13. 10.1073/pnas.1017547108 21173249 PMC3017162

[35] Khokha R, Murthy A, Weiss A. Metalloproteinases and their natural inhibitors in inflammation and immunity. Nat Rev Immunol. 2013; 13:649–65. 10.1038/nri3499 23969736

[36] Wang F, Yang H, Deng Z, Su Y, Fang Q, Yin Z. HOX Antisense lincRNA HOXA-AS2 Promotes Tumorigenesis of Hepatocellular Carcinoma. Cell Physiol Biochem. 2016; 40:287–96. 10.1159/000452545 27855366

